# SUCCESSFUL COMPLIANCE OF FAMILY CAREGIVERS IN DEMENTIA RESEARCH

**DOI:** 10.1093/geroni/igad104.1975

**Published:** 2023-12-21

**Authors:** Henrietta Armah, Vicki Winstead, Mustafa Yildiz, Jasmine Vickers, Carolyn Pickering

**Affiliations:** University of Alabama at Birmingham, Birmingham, Alabama, United States; University of Alabama at Birmingham, Birmingham, Alabama, United States; University of Alabama at Birmingham, Birmingham, Alabama, United States; University of Alabama at Birmingham, Birmingham, Alabama, United States; University of Alabama at Birmingham, Birmingham, Alabama, United States

## Abstract

Attrition is an issue for all longitudinal studies. One concern with intensive protocols with many repeated measures is that participants will drop out before completing the protocol, or will not complete enough of the surveys to provide meaningful data. Features of the study design can be important in increasing compliance with study participations. These features include incentives, survey windows, clear direction, ease of use of the data collection platform, and reminder protocols. This presentation examines compliance, defined as completion of >50% of all surveys, among 3 different micro-longitudinal daily diary studies. Compliance was high for all three studies (78%, 88%, 89%). The lowest compliance rate of 78% was an 8-day protocol with four diaries each day; three diaries were sent out randomly each of the eight days and the one nightly recap was sent at the same time each night. This suggests having multiple surveys in a single day reduce compliance with the study protocol. Compliance did not vary by other study features such as incentives, reminder method or length of the study. For daily diary studies, findings suggest that by doing once-a-day surveys, data collection protocols can be longer and total incentives ranging from $82-$91 are acceptable for maintaining high compliance.

